# Does consensus contours improve robustness and accuracy on $^{18}$F-FDG PET imaging tumor delineation?

**DOI:** 10.1186/s40658-023-00538-7

**Published:** 2023-03-13

**Authors:** Mingzan Zhuang, Zhifen Qiu, Yunlong Lou

**Affiliations:** Department of Nuclear Medicine, Meizhou People’s Hospital, Meizhou, China

**Keywords:** Consensus contours, Tumor delineation, Accuracy, Robustness, $^{18}$F-FDG PET imaging

## Abstract

**Purpose:**

The aim of this study is to explore the robustness and accuracy of consensus contours with 225 nasopharyngeal carcinoma (NPC) clinical cases and 13 extended cardio-torso simulated lung tumors (XCAT) based on 2-deoxy-2-[$^{18}$F]fluoro-D-glucose ($^{18}$F-FDG) PET imaging.

**Methods:**

Primary tumor segmentation was performed with two different initial masks on 225 NPC $^{18}$F-FDG PET datasets and 13 XCAT simulations using methods of automatic segmentation with active contour, affinity propagation (AP), contrast-oriented thresholding (ST), and 41% maximum tumor value (41MAX), respectively. Consensus contours (ConSeg) were subsequently generated based on the majority vote rule. The metabolically active tumor volume (MATV), relative volume error (RE), Dice similarity coefficient (DSC) and their respective test–retest (TRT) metrics between different masks were adopted to analyze the results quantitatively. The nonparametric Friedman and post hoc Wilcoxon tests with Bonferroni adjustment for multiple comparisons were performed with $P<$ 0.05 considered to be significant.

**Results:**

AP presented the highest variability for MATV in different masks, and ConSeg presented much better TRT performances in MATV compared with AP, and slightly poorer TRT in MATV compared with ST or 41MAXin most cases. Similar trends were also found in RE and DSC with the simulated data. The average of four segmentation results (AveSeg) showed better or comparable results in accuracy for most cases with respect to ConSeg. AP, AveSeg and ConSeg presented better RE and DSC in irregular masks as compared with rectangle masks. Additionally, all methods underestimated the tumour boundaries in relation to the ground truth for XCAT including respiratory motion.

**Conclusions:**

The consensus method could be a robust approach to alleviate segmentation variabilities, but did not seem to improve the accuracy of segmentation results on average. Irregular initial masks might be at least in some cases attributable to mitigate the segmentation variability as well.

## Introduction

In recent years, the delineation of the tumor boundary in positron emission tomography (PET) imaging (such as the primary tumor or large metastasis lesions) is increasingly crucial in radiation treatment planning, tumor response and prognosis [1–3]. However, it is difficult to distinguish the tumor boundary from noisy PET images. Although a large number of PET segmentation methods have been developed during the last 20 years, the validation of most published algorithms is either insufficient or inconsistent [4]. Daisne et al. [1] showed the potential value of PET imaging for the tumor delineation in head and neck cancers on condition that a proper segmentation method is applied. Nestle et al. [5] compared different delineation methods in PET imaging for patients with non-small cell lung cancer, and found that different methods resulted in substantially different tumor contours and required further evaluation with patient data. It seems that the tumor volume delineation in PET imaging is method-dependent and sensitive to high inter and intra-operator variability, presenting special challenges to obtain quantitative metrics consistently and accurately [6].

In view of the foregoing, a method for tumor volume delineation in PET imaging which is non-sensitive against various imaging situations and could be simply used in clinical routine is highly demanding. The decision of the best segmentation method seems to be highly subject to the imaging procedures, inter and intra-operator conditions [4, 6, 7]. Therefore, using consensus contours based on different individual segmentation results, may be the solution for PET imaging segmentation against various clinical situations. Lv et al. [8] used the intersections of two manual segmentations to derive radiomics features and assessed their prognostic performance for nasopharyngeal carcinoma (NPC) patients. Cao et al. [9] explored the potential applications of dose painting using PET/MR for NPC patients and discovered volume contours from different images were varied and the volume determined by cluster-analysis might be considered in radiation oncology. McGurk et al. [10] investigated the use of combining segmentations to reduce the various performances of different segmentation methods, and found combining segmentations could improve accuracy and were robust against the varying performances of different segmentation methods. Schaefer et al. [11] evaluated the influence of consensus methods on different segmentations and discovered that consensus contours could offers robustness against the inconsistent performance of different segmentation methods.

Despite that, it is still questioned to the absence of realistic simulated ground truth and the limited size of clinical data in these studies [10, 11]. Either simple phantom simulations with homogeneous activity levels or limited clinical data (< 40 patients) with manual delineation, CT or macroscopic specimen as the ground truth, were employed in these studies, which may not sufficiently demonstrate the robustness and accuracy of consensus contours. How the consensus methods perform with more realistic simulated tumors or a larger clinical patient data is still unclear. In this context, the purpose of our study was to validate the robustness and accuracy of consensus contours based on different segmentation methods through anthropomorphic phantom simulation and a much larger clinical database in 2-deoxy-2-[$^{18}$F]fluoro-D-glucose ($^{18}$F-FDG) PET imaging.

## Methods

### Anthropomorphic phantom simulation

In our study, realistic anthropomorphic phantom simulations were constructed from the extended cardio-torso (XCAT) phantom as described previously [12, 13]. Specifically, realistic tumor shapes were derived from clinical data (7 lung tumors and 6 cases of laryngeal squamous cell carcinoma) by thresholding methods and integrated into the XCAT phantom following the approaches proposed by Le Maitre et al. [14, 15]. In our study three different activity levels were modeled within the tumor to simulate the realistic intratumoral uptake heterogeneity. Then, several $^{18}$F-FDG time-activity curves (TACs) were generated based on two-tissue compartment model and kinetic parameters provided in the literature (Table 2)[13, 16–20]. Each modeled TAC was appointed to the corresponding tissue in the simulation phantom. Finally, the noise-free simulated emission maps were generated at 70 min post injection, and the levels of the uptake ratio within the lesions at 70 min were 11.01:10.14:6.62, with the background outside the lesions set to 1.

**Table 1:** Patient characteristics (n=225)

Characteristics	
Patient	225
Men	150
Age (y)	50 (43, 58)
*Tumor type (histology)*	
Undifferentiated non-keratinizing	221
Differentiated non-keratinizing	2
Poorly differentiated keratinizing	2
Tumor stage	
I	6
II	35
III	100
IV	84
Injected activity (MBq)	280.83 (249.38, 313.02)
Weight (kg)	57 (51, 64)
Range of MATV (cm$^3$)	10.95 (6.72, 19.86)

Furthermore, to assess the influence of respiratory motion, the simulation was also conducted with and without respiratory motion, respectively. Specifically, a 5 s breathing cycle with maximum diaphragm motion of 1.5 cm, maximum anterior-posterior expansion of 0.5 cm was adopted and divided into 10 bins. Then, the noise-free simulation including respiratory motion was finalized by taking the average of these 10 bins assuming no uptake activity change during the breathing cycle of 5 s, and the static simulation was produced at the intermediate time point of respiratory cycle (bin 3).

**Fig. 1:**
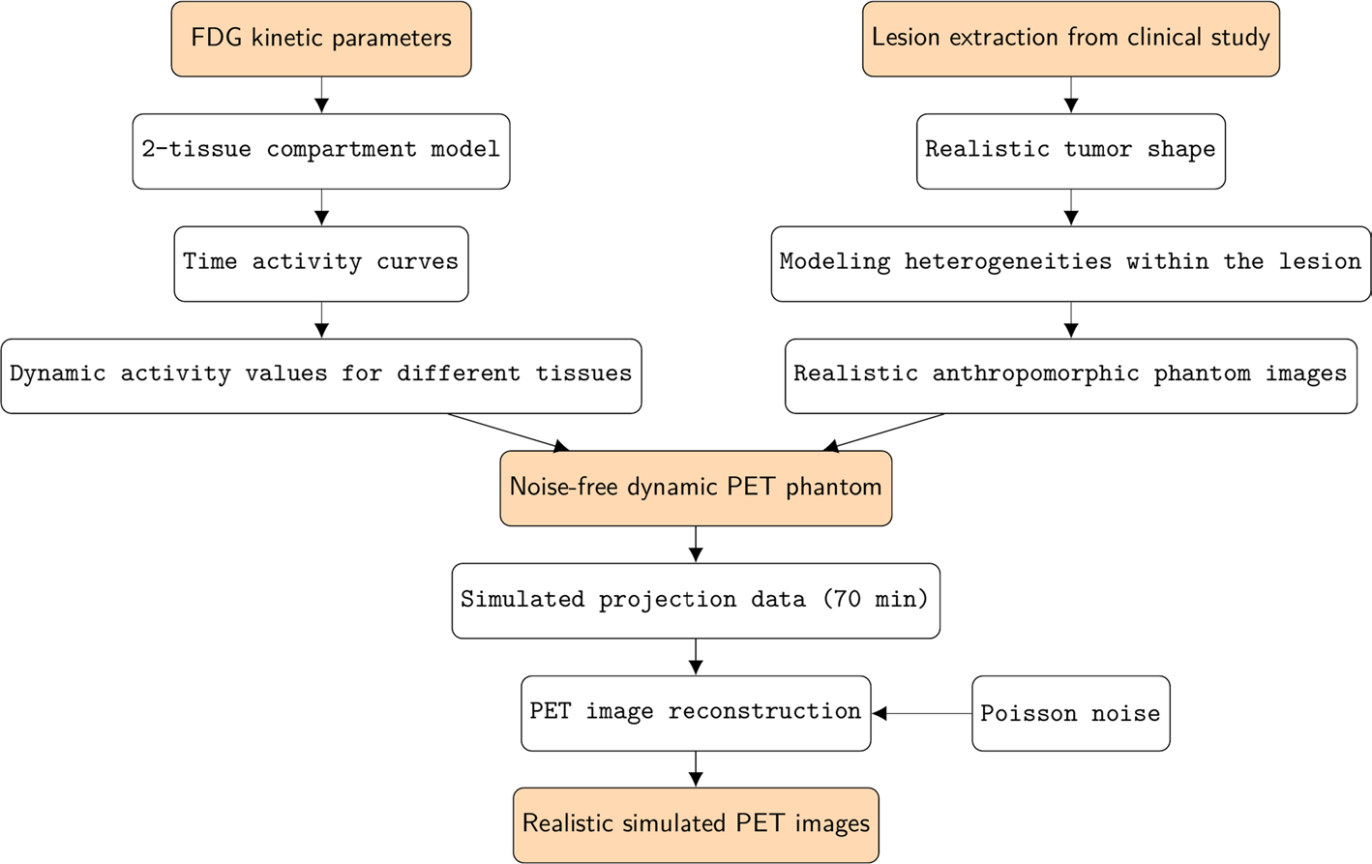
Flowchart showing various steps in the simulation of the realistic anthropomorphic model

At last, an analytical fully 3D forward projector was adopted to generate the PET sinograms based on the model of a Siemens Biograph mCT PET/CT scanner. Subsequently, the corresponding attenuation maps were applied to the sinograms to get the attenuated PET data and quantitative levels of Poisson noise were later added, which were equivalent to a 180 s acquisition time per bed. The noisy projection data were then reconstructed using an ordered subsets expectation maximization (OSEM) algorithm with 14 subsets and 28 sub-iterations, followed by application of a Gaussian smoothing filter of 2 mm. All these projection and reconstruction procedures were performed within the software for tomographic image reconstruction (STIR) [21]. The matrix size of all simulated images was $200 \times 200$ with a voxel size of $0.50 \times 0.41 \times 0.41~{\text {cm}}^3$. In total, 13 different tumors including the range of respiratory motion (XCAT$_{av}$, volume range 6.64–69.34 cm$^3$) and without respiratory motion (XCAT$_{st}$, volume range 3.57–54.66 cm$^3$) in the lung location were simulated. After the simulating with the introduction of noise, the ratios between the mean activity within the tumor (simulated ground truth) and the background mask for XCAT$_{av}$ and XCAT$_{st}$ are 3.44 and 4.04, respectively. The simulation flowchart is shown in Fig. 1.

### Clinical database

The clinical studies include 225 cases of NPC, for which whole-body $^{18}$F-FDG PET images were acquired using an mCT PET/CT scanner (Siemens, Germany) at Meizhou people’s hospital from 2018 to 2020. The ground truth in clinical studies is not known a priori, which makes it infeasible to assess the accuracy of segmentation results but possible to investigate the robustness of different segmentation methods with different tumor masks. All patients fasted for at least 6 h before $^{18}$F-FDG PET/CT imaging. According to the patients’ body weight (4.81 MBq/kg), $^{18}$F-FDG was injected and PET/CT scanning was performed after 60 min uptake. PET data were then reconstructed using a vendor-provided TrueX algorithm (21 subsets and 2 iterations) with time-of-flight, using low-dose CT for attenuation correction, and the matrix size of all reconstructed images was 200 $\times$ 200 resulting in a voxel size of 4.07 mm $\times$ 4.07 mm $\times$ 3.00 mm. A post-reconstruction Gaussian smoothed filter with 5 mm full-width at half-maximum was also applied to PET data. Patient characteristics are presented in Table 1. The mean uptake ratio within the tumor (segmentation results) and the background mask is 2.60. This study was approved by Meizhou people’s hospital ethics committee.

**Table 2:** $^{18}$F-FDG kinetic parameters used to generate time-activity curves in the simulation study

Tissue	$K_1$ (ml/(min*g))	$k_2$ (l/min)	$k_3$ (l/min)	$k_4$ (l/min)	$V_B$ (ml/ml)
Level I in lesion	0.180	0.990	0.190	–	0.036
Level II in lesion	0.150	0.550	0.120	–	0.071
Level III in lesion	0.110	0.400	0.073	–	0.095
Normal lung	0.108	0.735	0.016	0.013	0.017
Normal liver	0.864	0.981	0.005	0.016	–
Myocardium	0.600	1.200	0.100	0.001	–
Normal bone marrow	0.200	0.680	0.050	0.020	0.010

### PET segmentation and analysis

To assess the impact of tumor delineation, four different segmentation methods were applied: a method for automatic segmentation using an active contour model (MASAC) [12] and an affinity propagation algorithm (AP) [22], the contrast-oriented thresholding method (ST) of Schaefer et al. [23], and segmentation using 41% of the maximum tumor value as a threshold (41MAX) [24]. Specifically, the parameter lambda in MASAC was set to 3 while the default parameters were kept for AP with the largest grouping as its segmentation result.

For each case, either the simulated lung tumor or the primary tumor in clinical data, these four different segmentation methods were employed to delineate the tumor volume automatically. Furthermore, each segmentation method was performed with two different user-defined tumor masks (a regular rectangle area, and an irregular cropping area) to assess the robustness of segmentation methods. The consensus segmentation method (ConSeg), by applying the majority vote rule based on four different segmentation results, is adopted in our study [10, 11].

The metabolically active tumor volume (MATV) was taken for quantitative assessment of segmentation results. For simulated cases with known ground truth, the relative volume error (RE) and Dice similarity coefficient (DSC) were also adopted to assess the accuracy of these PET segmentation methods quantitatively, which are defined as below:

1 $$\begin{aligned} RE= & {} \frac{(MATV(SM)-MATV(GT))}{MATV(GT)}\times 100\% \end{aligned}$$

2 $$\begin{aligned} DSC= & {} \frac{2\times \left| SM\cap GT \right| }{\left| SM \right| +\left| GT \right| } \end{aligned}$$

where *SM* and *GT* represent the segmentation method and the ground truth, respectively [12, 25–27]. Besides, to assess the robustness of different methods, the test–retest reproducibility of the metrics between two different initial tumor masks ($TRT_{Metrics}$) was calculated as:

3 $$\begin{aligned} TRT_{Metrics} = \frac{{Metrics_{rectangle}\mathrm{{ - }}Metrics_{irregular}}}{{(Metrics_{rectangle}\mathrm{{ + }}Metrics_{irregular})/2}} \end{aligned}$$

where $Metrics_{rectangle}$ and $Metrics_{irregular}$ are metrics derived from rectangle masks and irregular cropping masks, respectively.

### Statistical analysis

Statistical analysis was performed using R 4.1.3 software [28]. The nonparametric Friedman and post hoc Wilcoxon tests with Bonferroni adjustment for multiple comparisons were performed to assess the difference among different segmentation results. A *P* value < 0.05 was considered to be significant. The results are expressed as median with inter-quartile range (IQR) in parentheses, and presented as box-and-whisker plots as appropriate, providing lower to upper quartile (25 to 75 percentile, central box), the median (middle line of the box) and the minimum to maximum value. Specifically, the minimum in plots is calculated as the lower quartile minus 1.5*IQR and the maximum is calculated as the upper quartile plus 1.5*IQR. For display purposes, outliers identified as 1.5*IQR were removed from plot (whiskers).

## Results

### Representation segmentation results

**Fig. 2:**
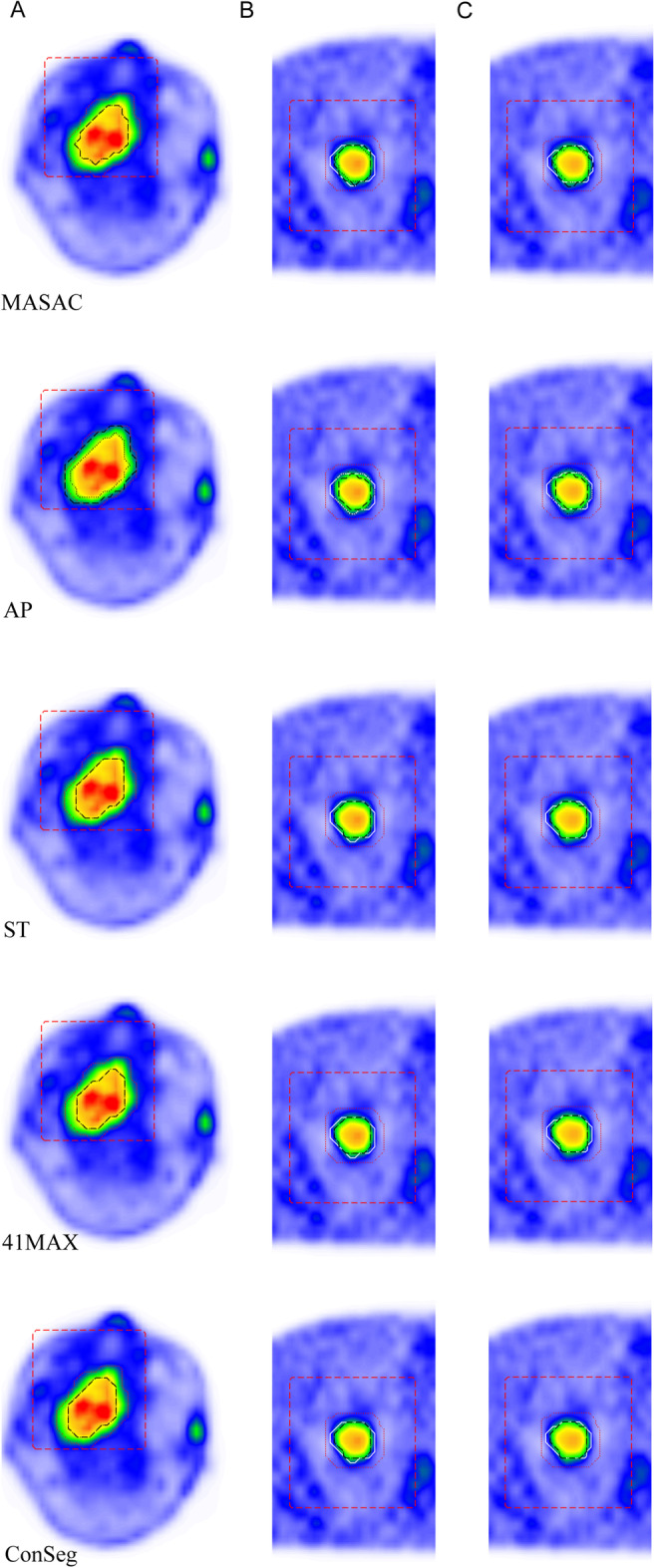
Representative contours of five different methods (black dashed horizontal line: contours with the rectangle mask, black dotted line: contours with the irregular cropping mask, white:*simulated ground truth *) with two initial masks (red dashed horizontal line: rectangle mask, red dotted line: irregular cropping mask) in clinical database (**A**) , anthropomorphic simulation with **B** and without** C** respiratory motion

The evaluated data in this study involved NPC clinical database and XCAT anthropomorphic phantom simulation. Fig. 2 showed different delineated contours by different methods in NPC (A), XCAT$_{av}$ (B) and XCAT$_{st}$ (C). It could be observed that AP showed higher variability under different initial masks as compared to other methods and enhanced accuracy in irregular masks compared with rectangle masks. Besides, it seems obvious that all methods underestimated the lesion to segment in XCAT$_{av}$.

### Robustness test

**Fig. 3:**
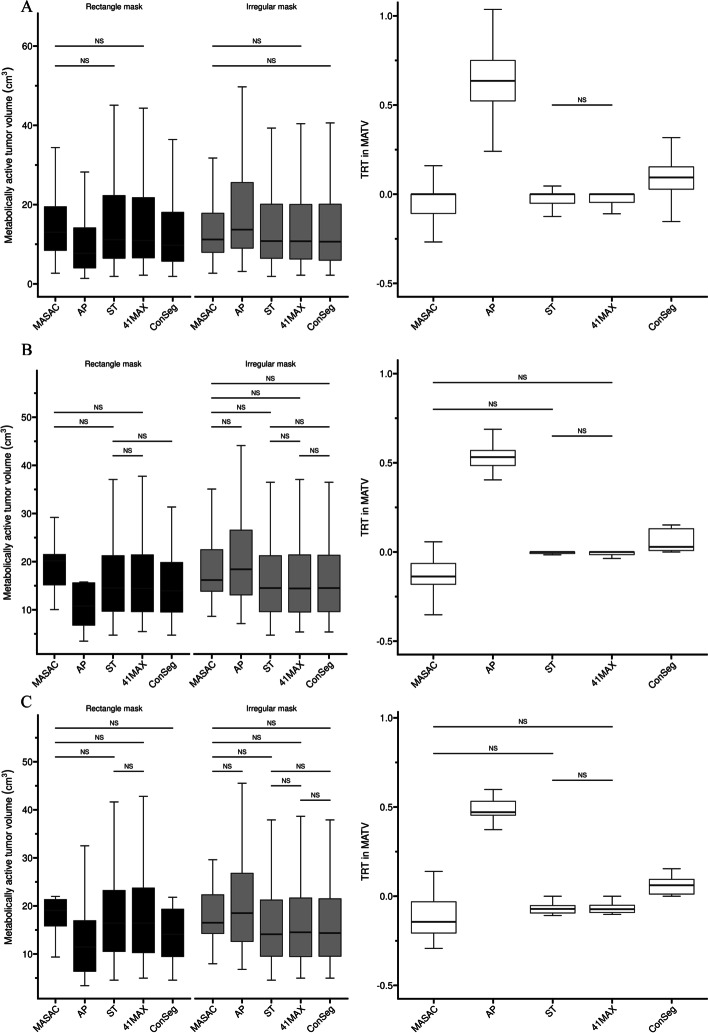
Box-and-whisker plots of MATV and $TRT_{MATV}$ for each method in clinical database (**A**) and anthropomorphic simulation with (**B**) and without** C** respiratory motion. For display purposes, outliers identified as 1.5*interquartile range were removed from plot (whiskers). Comparisons without statistically significant differences are marked with horizontal line

As can be seen from Fig. 3, the use of different masks caused significantly different segmentation results in MATV for both evaluated data and most segmentation methods except ST in XCAT$_{av}$ (*P* = 0.059) and MASAC in XCAT$_{st}$ (*P* = 0.080). Specifically, for either NPC or XCAT, AP presented the highest variability for MATV with the use of different masks with respect to other segmentation methods. The use of rectangle masks resulted in a significantly smaller MATV (NPC: $-$43.68%, XCAT$_{av}$: $-$41.44% and XCAT$_{st}$: $-$38.10%) for AP compared with irregular masks. Besides, ST and 41MAX showed relatively consistent performances for MATV in different masks and there was no significant difference in $TRT_{MATV}$ between ST and 41MAX (Fig. 3 and Tables 3 and 4).

**Table 3:** Quantitative metrics used for assessment of the four individual segmentation methods, the average of four segmentation results (AveSeg) and the consensus method (ConSeg) for the simulated phantom studies including respiratory motion

Method	MATV(cm$^3$)	RE(%)	DSC
*Rectangle mask*			
MASAC	20.15 (15.18, 21.48)	− 18.36 (− 40.52, 5.24)	0.79 (0.74, 0.86)
AP	10.78 (6.80, 15.59)	− 54.27 (− 57.38, − 50.36)	0.63 (0.60, 0.65)
ST	14.51 (9.70, 21.23)	− 38.76 (− 41.79, − 35.03)	0.75 (0.73, 0.77)
41MAX	14.43 (9.62, 21.40)	− 38.28 (− 41.74, − 33.87)	0.76 (0.73, 0.77)
AveSeg	14.83 (11.38, 19.91)	− 41.61 (− 42.65, − 31.03)	0.73 (0.72, 0.74)
ConSeg	13.93 (9.54, 19.82)	− 42.95 (− 45.06, − 41.96)	0.73 (0.71, 0.73)
*Irregular mask*			
MASAC	16.17 (13.85, 22.48)	− 35.17 (− 45.10, − 17.74)	0.78 (0.71, 0.83)
AP	18.41 (13.10, 26.54)	− 22.67 (− 26.63, − 20.60)	0.83 (0.82, 0.85)
ST	14.51 (9.62, 21.23)	− 38.76 (− 41.79, − 36.05)	0.75 (0.74, 0.77)
41MAX	14.43 (9.54, 21.40)	− 38.28 (− 42.00, − 35.03)	0.76 (0.73, 0.78)
AveSeg	15.88 (11.47, 22.91)	− 35.83(− 37.21, − 30.53)	0.77 (0.77, 0.78)
ConSeg	14.51 (9.62, 21.32)	− 38.52 (− 41.74, − 36.05)	0.76 (0.74, 0.78)

**Table 4:** Quantitative metrics used for assessment of the four individual segmentation methods, the average of four segmentation results (AveSeg) and the consensus method (ConSeg) for the simulated phantom studies without respiratory motion

Method	MATV(cm$^3$)	RE(%)	DSC
*Rectangle mask*			
MASAC	19.08 (15.84, 21.32)	21.69 (− 22.04, 45.80)	0.84 (0.81, 0.86)
AP	11.45 (6.39, 16.92)	− 27.41 (− 34.27, − 25.73)	0.80 (0.75, 0.82)
ST	16.42 (10.53, 23.22)	− 6.98 (− 9.71, 4.90)	0.85 (0.82, 0.87)
41MAX	16.42 (10.28, 23.72)	− 4.98 (− 9.45, 8.82)	0.86 (0.83, 0.88)
AveSeg	15.57 (10.76, 21.05)	− 2.35 (− 15.70, 0.00)	0.83 (0.79, 0.86)
ConSeg	14.10 (9.46, 19.32)	− 15.88 (− 21.67, − 10.05)	0.87 (0.85, 0.88)
*Irregular mask*			
MASAC	16.51 (14.27, 22.31)	1.81 (− 26.32, 28.35)	0.84 (0.82, 0.87)
AP	18.50 (12.61, 26.79)	16.03 (4.90, 21.18)	0.89 (0.87, 0.92)
ST	14.10 (9.54, 21.23)	− 14.95 (− 15.93, − 5.85)	0.89 (0.86, 0.91)
41MAX	14.51 (9.46, 21.65)	− 12.98 (− 14.69, − 5.85)	0.88 (0.86, 0.91)
AveSeg	15.61 (11.47, 23.00)	− 0.40 (− 13.20, 6.14)	0.88 (0.86, 0.90)
ConSeg	14.35 (9.54, 21.48)	− 12.21 (− 15.73, − 5.85)	0.88 (0.86, 0.91)

**Fig. 4:**
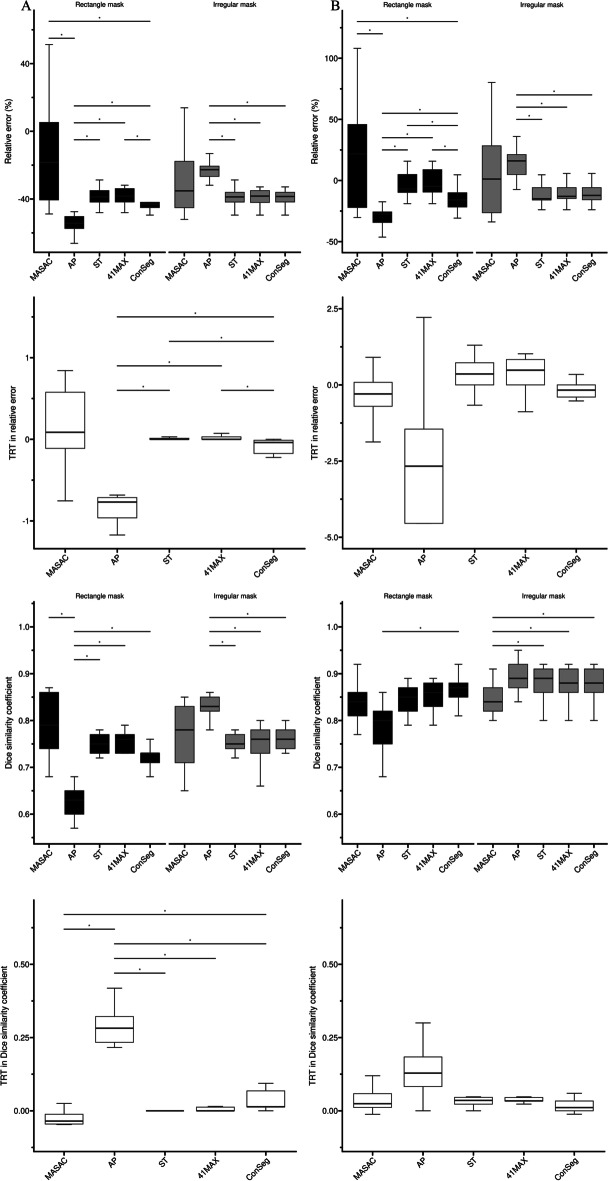
Box-and-whisker plots of RE, DSC and their respective TRT metrics for each method in the anthropomorphic simulation with (**A**) and without** B** respiratory motion. For display purposes, outliers identified as 1.5*interquartile range were removed from plot (whiskers). Statistically significant differences are marked with horizontal line

It should be noted in Fig. 3 that for both evaluated data ConSeg presented much better TRT performances in MATV (NPC: 0.09, XCAT$_{av}$: 0.03 and XCAT$_{st}$: 0.06) compared with AP (NPC: 0.64, XCAT$_{av}$: 0.53 and XCAT$_{st}$: 0.47), and slightly poorer TRT in MATV compared with ST or 41MAX in most cases (NPC: 0.00, XCAT$_{av}$: 0.00 and XCAT$_{st}$: $-$0.07). Similar trends were also found in RE and DSC with the simulated data (Fig. 4), where AP showed the poorest TRT performances (RE: $-$0.77 in XCAT$_{av}$ and $-$2.67 in XCAT$_{st}$, DSC: 0.28 in XCAT$_{av}$ and 0.13 in XCAT$_{st}$) and ConSeg achieved much better TRT (RE: $-$0.04 in XCAT$_{av}$ and $-$0.17 in XCAT$_{st}$, DSC: 0.01 in XCAT$_{av}$ and XCAT$_{st}$) with respect to AP.

### Accuracy test

Applying the consensus method seemed to alleviate the high variability of different segmentation results, but did not improve the segmentation accuracy on average (Tables 3 and 4 and Fig. 4). More specifically, the average of four segmentation results (AveSeg) showed slightly better RE (rectangle: 3.12%, irregular: 6.98%) in XCAT$_{av}$ and much better RE (rectangle: 85.20%, irregular: 96.72%) in XCAT$_{st}$ with respect to those in ConSeg. Besides, compared with ConSeg, AveSeg presented no significant difference for rectangle masks and slightly better DSC for irregular cases in XCAT$_{av}$ (AveSeg: 0.77, ConSeg: 0.76), but mildly poorer DSC for rectangle masks (AveSeg: 0.83, ConSeg: 0.87) and comparable DSC for irregular cases in XCAT$_{st}$.

Furthermore, AveSeg and ConSeg both presented much improved RE from 10.31% to 82.99% and slightly increased DSC from 1.15% to 6.02% in irregular masks as compared with rectangle masks (Tables 3-4). AP also displayed much better segmentation results in XCAT$_{av}$ (RE: 58.23%, DSC: 31.75%) and XCAT$_{st}$ (RE: 41.52%, DSC: 11.25%) with the use of irregular masks than rectangle masks. Besides, MASAC presented a much larger IQR for RE or DSC in either rectangle or irregular masks compared with other segmentation methods in most cases except for DSC in XCAT$_{st}$. Additionally, it is apparent that all methods underestimated the tumour boundaries in relation to the ground truth for XCAT simulated data including respiratory motion.

## Discussion

It has been substantiated that large variability in different segmentation results was presented and all segmentation needed to be reviewed thoroughly [4, 6]. To reduce these variability, the consensus method was widely adopted in many study [10, 11, 29, 30]. Our study showed that quantitative metrics, such as MATV, DSC and RE, could be highly influenced by different segmentation methods and initial cropping masks, and the use of consensus contours could help to mitigate certain individual methods that may have high segmentation variability under different conditions. In other words, the consensus method could alleviate these variability and enhance the robustness of tumor segmentation, but it did not seem to improve the segmentation accuracy on average.

Our results seem inconsistent with the study of McGurk et al. [10], in which only homogeneous activity levels were simulated with the National Electrical Manufacturers Association image quality phantom, and it was found that improved segmentation accuracy was achieved by the consensus method compared with any one individual method for all simulated shapes, sizes, contrasts, and scan durations. This is because the consensus method in our study, taking the majority of different independent results as consensus contours, was still influenced by different individual segmentation results. For example, if all individual segmentation methods underestimate or overestimate the lesion to contour, it is reasonable for the consensus method to have an average bias on the segmentation results. Besides, in theory the majority approach could improve the accuracy of segmentation results if the accuracy of each individual segmentation method is greater than 0.5. However, because of the diversity of PET images depending on clinical indications, it is difficult for each segmentation method to have accuracy greater than 0.5 for all types of clinical oncology indications and so the accuracy of the majority vote method might not be improved relative to the individual segmentation method on average.

The ground truth for segmentation in clinic is difficult to achieve. In our study, we constructed the anthropomorphic phantom simulation based on our previous work [13] and found that most methods, with the exception of AP in rectangle mask, achieved a median DSC greater than 0.7, indicating good segmentation agreement [10, 26, 31, 32]. However, it should be noted that all methods apparently underestimated the simulated ground truth including the range of respiratory motion on average (Rectangle Mask: $-$41.61%, Irregular Mask: $-$35.83%), whereas the average of the segmentation in the simulation without respiratory motion showed $-$2.35% for rectangle masks and $-$0.40% for irregular masks. It is known that the respiratory motion would amplify the actual static ground truth and blur the tumor boundary, and so it is reasonable for the segmentation methods to generate the contours much smaller than the simulated ground truth with its range of respiratory motion included. Therefore, in routine clinical practice the respiratory motion and its blurring effect on PET segmentation and quantitative analysis should be carefully considered. The use of gating acquisition and positioning immobility masks might also help to reduce the impact of motion during PET scanning. motion.

For most segmentation approaches, manual interaction is often required during the segmentation process [33, 34]. Although large variability exists in different segmentation results for both initial masks, the consensus method showed robustness against the inconsistent performance of individual segmentation methods, consistent with the results found by Schaefer et al. [11]. Further to this, segmentation results with irregular cropping area seem less varied than those with regular rectangle masks, suggesting manually contoured initial masks might be at least in some cases attributable to mitigate this variability.

Different kinds of image characteristics may have different quality images. Providing a good quality images could be acquired by different data acquisition/reconstruction protocol, it would be possible to mitigate the variability in target volume segmentation. For example, the dynamic Patlak-derived net uptake rate constant (Ki) PET imaging has also been proposed in the literature, which could track the four-dimensional (4D) distribution of the tracer uptake post-injection quantitatively and may improve the lesion detectability in the clinic [13, 35–37]. Xiang et al. [38] compared $^{18}$F-FDG and $^{18}$F-FLT PET/CT images in gross target volume delineation on VX2 rabbit model and found $^{18}$F-FLT PET/CT could present the tumor boundaries more accurately. The implementation of the AAPM report No. 174 recommendations for $^{18}$F-FDG PET imaging in radiation therapy, such as staging, segmentation, image registration, treatment planning, and therapy response assessment [39], as well as the publication of strict PET imaging guidelines [40, 41], may also help to alleviate the variability in image segmentation.

One of the limitations in our study is the absence of ground truth data for NPC clinical dataset, which prevents the assessment of the accuracy of MATV segmentation in clinic. Furthermore, the presented simulated data may not be sufficient to fully demonstrate the bias of segmentation results with respect to treatment assessment in clinical setting. Therefore, our results should be validated with a benchmark using harmonized and standardized data in the future [42]. The use of digital PET or long axial field of view PET scanners might also be helpful to improve tumor segmentation and analysis, thereby mitigate these variabilities. In addition, we did not investigate the time-phase effect of $^{18}$F-FDG uptake on segmentation results in our study. Chen et al. [43] evaluated changes in SUV values of $^{18}$F-FDG PET/CT on NPC tumor volume with 8 delineation methods and recommended the anatomic biologic contouring between 35 and 55 min after injection as the first choice for tumor delineation. We should acknowledge that the uptake changes would not only impact SUV values, but also affect other PET image characteristics, such as texture pattern, which is not evaluated in our study.

## Conclusions

Quantitative results derived from our segmentation studies on 13 realistic simulated tumors and 225 NPC clinical data show that although quantitative metrics could be highly influenced by different segmentation methods and initial cropping masks, the consensus method could help to alleviate the variability of individual segmentation results under different conditions and enhance the robustness of tumor segmentation, but did not seem to improve the accuracy of segmentation results on average relative to the individual segmentation method. Irregular cropping initial masks might be at least in some cases attributable to mitigate the segmentation variability as well.

## Data Availability

The datasets used and analysed during the current study are available from the corresponding author on reasonable request.
